# Comprehensive investigation identifies CPSF3 as a novel prognostic and oncogenic biomarker in bladder cancer

**DOI:** 10.1007/s12672-025-03672-z

**Published:** 2025-10-10

**Authors:** Zhiyang Ma, Yining Hao, Wei He, Xin Xie, Danfeng Xu, Chenghe Wang

**Affiliations:** Department of Urology, Ruijin Hospital, Shanghai Jiao Tong University School of Medicine, 197 Ruijin Second Road, Shanghai, 200025 P.R. China

**Keywords:** Bladder cancer, CPSF3 protein, Immunohistochemistry, Prognosis

## Abstract

**Background:**

Bladder cancer (BC) remains a prevalent malignancy worldwide, with rising incidence rates each year. Despite progress in therapeutic strategies, many patients suffer recurrence or progression, emphasizing the urgent need for novel prognostic biomarkers and therapeutic targets. This research evaluated the prognostic relevance and functional role of Cleavage and Polyadenylation Specificity Factor 3 (CPSF3) in BC.

**Methods:**

We analyzed CPSF3 expression using The Cancer Genome Atlas data and immunohistochemistry on a cohort of 203 BC patients. A nomogram incorporating CPSF3 expression was developed based on CPSF3 expression for prediction of overall survival and disease-free survival. Immune infiltration analyses and transcriptome sequencing were performed to explore underlying biological mechanisms. In vitro and in vivo experiments were utilized to examine the results of CPSF3 silencing on bladder cancer cell growth, colony-forming ability and cell cycle transitions.

**Results:**

Elevated CPSF3 expression was significantly linked to unfavorable overall survival and disease-free survival both in TCGA datasets and our cohort. The CPSF3-based nomogram outperformed conventional prognostic models. CPSF3 expression was associated with tumor-infiltrating immune cells and immune checkpoint markers. Enrichment analysis revealed CPSF3 enrichment in cell cycle-related pathways. Suppression of CPSF3 expression led to marked reductions in cell proliferation, colony formation, tumor growth in animal models and inhibited G1 to S phase progression.

**Conclusion:**

CPSF3 is a promising prognostic biomarker for BC and may play a crucial role in BC progression. Incorporating CPSF3 into clinical prognostic models may enhance prediction of patient outcomes. CPSF3 may represent a promising therapeutic target for BC management.

**Supplementary Information:**

The online version contains supplementary material available at 10.1007/s12672-025-03672-z.

## Introduction

Bladder cancer (BC) is a serious public health issue all over the world. Ranked as the 10th most prevalent cancer globally, it is estimated that approximately 82,290 new cases diagnosed each year [1]. Depending on the localization to the mucosa or submucosa, BC is devided into non-muscle-invasive bladder cancer (NMIBC) or muscle-invasive bladder cancer (MIBC) [2]. For patients with MIBC, the standard treatment is radical cystectomy (RC) [3]. Neoadjuvant therapy is also effective in certain circumstances. Despite diverse therapeutic approaches tailored to individual cases, a large number of BC patients suffer either a recurrent stuation or progression after treatment, which imposes a considerable societal burden [4–6]. Consequently, it is urgent to delve deeper into the mechanisms on the molecular level driving BC development and to explore new prognostic biomarkers or therapeutic targets that could enhance the prediction and management of BC.

The maturation of precursor mRNA (pre-mRNA) relies heavily on the crucial functions of 3’-end cleavage and polyadenylation [7], which are intricately connected to the subsequent processes of alternative splicing and alternative polyadenylation of RNA [8]. The key cis-acting element in the polyadenylation signal, which determines the cleavage and polyadenylation site, is the hexanucleotide sequence AAUAAA, usually found about 10–30 nucleotides upstream from the site of endonucleolytic cleavage [9–11]. When the transcriptional termination stage reaches and the consensus AAUAAA polyadenylation signal is recognized, pre-mRNA undergoes cleavage and polyadenylation. These steps are meticulously overseen by dedicated protein complexes to ensure both efficiency and precision. Cleavage and polyadenylation specificity factor 3 (CPSF3), as a member of CPSF complex, functions as an essential role of the pre-mRNA cleavage and polyadenylation machinery as well as providing sequence specificity [12, 13]. The interaction of CPSF3 with particular proteins leads to the formation of the processing complex essential for triggering its endonuclease function [14]. Although extensive research has been proceeded on the correlation between CPSF3 and the progression and prognosis of multiple cancers, to our best knowledge, the impact of CPSF3 on BC remains in-depth investigation.

Here, our aim was to explore the prognostic significance of CPSF3 in BC based on The Cancer Genome Atlas (TCGA; http://portal.gdc.cancer.gov) and used our cohort to further investigated its independently predictive capability on BC prognosis based on immunohistochemistry (IHC). Then we constructed a novel nomogram to assess whether CPSF3 was a superior prognostic marker for BC patients. Moreover, we employed RNA sequencing to investigate its potential biological roles of CPSF3 in BC, particularly its involvement in facilitating BC progression. Next, we conducted experiments in vitro and in vivo aiming to evaluate its molecular function in cell growth.

## Materials and methods

### Patient information and tissue samples

Our cohort included 203 patients diagnosed BC and underwent RC in Shanghai Jiao Tong University School of Medicine Affiliated Ruijin Hospital between May 2009 and May 2018, in which 161 patients had pairs of para-tumor/tumor samples and 42 patients with single tumor samples. Clinical data were updated in December 2022. This study got the approval from the Ethics Committee of Ruijin Hospital, School of Medicine, Shanghai Jiao Tong University (No.KY2022-183, 3rd August 2022). Patients participating in the study were noticed and agreed for the use of the samples and the publication of the clinical data. Formal consents were obtained. The experiment was conducted according to the ethical guidelines. All tumor and para-tumor samples were formalin-fixed and paraffin-embedded, diagnosed precisely by experienced pathologists. Patients’ medical records were comprehensively reviewed and all clinical information was extracted in an anonymous manner, including age, gender, smoking history, tumor grade, tumor size, tumor number, muscle invasion, vascular invasion, pTNM stage, AJCC stage, systemic therapy, follow-up period, status. pTNM stage was determined by 8th AJCC TNM staging system. AJCC stage was determined by 8th AJCC staging system. Systemic therapy was determined by whether the patient accepted any systemic therapy after RC. Overall survival (OS) was measured from the surgery date to death for any reason. Disease-free survival (DFS) was measured from the surgery date to disease recurrence for any reason.

### Public data acquisition and preprocessing

The RNA-sequencing (RNA-seq) data and corresponding clinical phenotype information were obtained from TCGA-BLCA datasets, containing results (FPKMs) of 411 BC samples and 19 normal bladder samples. mRNA was differentiated for next-step analysis, while lncRNA and miRNA were excluded. Finally we selected 406 independent tumor samples paired with follow-up and clinical information for further analyzing survival situation [15,16]. The genetic alteration features of CPSF3 was determined through the cBioPortal database (https://www.cbioportal.org/). The somatic mutation information of BLCA samples and CPSF3 expression was compared and visualized using the “maftools” package [17]. The IHC staining images of CPSF3 were downloaded from the HPA database (https://www.proteinatlas.org/) [18].

### IHC staining

Tumor samples from 203 patients and para-tumor samples from 161 of them were collected, formalin-fixed, paraffin-embedded and used to construct tissue microarrays (TMA). TMAs were placed in the oven and set the temperature to 63℃ for 1 h and transferred to the automatic staining machine for deparaffinization, including soaking in xylene for 15 min and then in gradient alcohols (100% for 7 min, 95%, 85% and 70% for 5 min). After that, TMAs were washed by distilled water three times and placed in the pressure cooker with boiled sodium citrate (pH = 6.0) for 5 min and after naturally cooling placed with boiled EDTA buffer for 20 min in order to retrieve antigen. Immuno staining was applied to block non-specific binding regions for 15 min. Following that, TMAs were washed using PBS buffer 3 times and added with primary antibody against human CPSF3 (1:50 dilution, rabbit, Abmart) overnight at 4℃ temperature. Next day TMAs were added with secondary antibody (rabbit) at 25℃ temperature for 30 min followed by reheating to 37℃ and washed by PBS buffer. Later, after PBS buffer washing, prepared DAB dilution was applied to TMAs and the staining intensity was observed for up to 5 min. Finally, TMAs were treated with Harris hematoxylin (SIGMA) for 1 min and then dehydrated in 0.25% hydrochloric acid alcohol for at least 2 s before sealing TMAs.

### Evaluation of CPSF3 staining intensity

The intensity of the staining was evaluated under a multihead microscope and quantified through H-score, which was calculated based on both the intensity of staining and the percentage of cells stained positively [19]. The intensity of staining was graded as 0 (no staining), 1+ (weak staining), 2+ (moderate staining), or 3+ (strong staining). The H-score was then computed using the following formula:

$$H-score=\mathrm{}pi(i+1)(i= 1,2,3)$$                                          

ImageJ software (version 1.54 g) and “IHC profiler” plugin were used to conduct the evaluation of the staining and then we calculated the final H-score.

### Immune-related cell infiltration analysis

To evaluate the immune cell infiltration level in the tumor microenvironment (TME), we analyzed the expression status of CPSF3 in diverse cell types according to dataset GSE149652 by TISCH2 database (http://tisch.comp-genomics.org/) [20, 21]. Then, 24 different immune cell types were included and “GSVA” package was employed to analyze RNA-seq data sourced from TCGA database [21, 22]. Spearman correlation test was conducted to investigate whether CPSF3 expression was correlated with the infiltration level of immune cells and common immune checkpoints.

### Cell culture and transfection

Human BC cell lines (J82, T24, UMUC3, EJ, and 5637) and normal uroepithelium cell line (SV-HUC-1) were obtained from ATCC. The details of the cell lines used in the experiments could be checked in Table S2. The cell lines were authenticated within three years prior to use. We have conducted mycoplasma testing on these cell lines and the results were all negative. T24, EJ and 5637 cell lines were cultured using RPMI 1640 medium (Gibco, USA). J82 cell lines were cultured using high glucose Dulbecco’s Modified Eagle’s medium (DMEM) (Gibco, USA). UMUC3 cell lines were cultured using minimal essential medium (MEM) (Gibco, USA). SV-HUC-1 cells were cultured using Ham’s F-12 K medium (Gibco, USA). All the medium was added with fetal bovine serum (FBS, Gibco, USA) to a concentration of 10%. Four small interfering RNA (siRNA) for negative control (NC) and CPSF3 were designed by GenePharma (Shanghai, China). RNAiMAX (Invitrogen, USA) was used to transfect T24 and UMUC3 cells with siRNA. CPSF3 mRNA expression was detected 24 h after transfection.

### Quantitative reverse-transcription polymerase chain reaction (qRT-PCR)

RNA-Quick Purification Kit (ShareBio, China) was used to extracting total RNA from BC cells. Then 1 µg RNA was reversed to cDNA using the First Strand cDNA Synthesis Kit (Beyotime, China) following manufacturer’s protocol. The relative mRNA expression level was tested through SYBR Green qPCR Mix (Beyotime, China), with GAPDH as the endogenous control. Primers needed in this study were designed and synthesized by BioSune (Shanghai, China) and the detail sequences were provided in Table S1.

### Western blotting

Total protein were extracted from BC cells using RIPA lysis buffer (NCM biotech, China). After separated by SDS-PAGE (10%) and then transmitted onto 0.22 μm PVDF membranes (Millipore, USA) which were then blocked with 5% skim milk. The membranes were washed with TBST buffer after blocking and incubated with primary antibodies overnight at 4℃ respectively. Primary antibodies were listed in Table S3. The next day, the washed membranes were incubated with goat anti rabbit HRP-conjugated secondary antibodies (Beyotime, China, A0208, 1:3000 dilution), protein expression levels were tested by SuperSignal West Attochemiluminescent substrate (Invitrogen, USA, A38554).

### Cell proliferation assay

The effect of CPSF3 on cell proliferation was tested by Cell Counting Kit-8 (CCK-8) assay. T24 and UMUC3 cells (5000 cells/well) were reseeded into 96-well plates 24 h after transfected with siRNA and si-NC. Cells were cultivated at 37℃ for 24, 48, 72, and 96 h. At every time point, the absorbance values at 450 nm was measured after each well were added with the mixture containing 10 µL CCK-8 (Vazyme, China) and 100 µL corresponding medium for 2 h.

### Colony formation assay

T24 and UMUC3 cells (1000 cells/well) were plated into six-well plates and cultured at 37℃ for 10 days. Next, the colonies were fixed with 4% paraformaldehyde fixation for 30 min and stained by 0.5% crystal violet (Beyotime, China) for 30 min. The colony number was calculated by ImageJ software(version 1.54 g).

### Flow cytometry

BC cells were collected 48 h after transfection and fixed with 75% ethanol overnight at 4℃. The next day, cells were added with RNase A and PI (Beyotime, China) for 30 min and washed with PBS. CytoFLEX flow cytometer (Beckman Counter, USA) were used to detect the distribution of cell cycle. Following RNase A/PI staining, single cells were gated by pulse-width versus pulse-area analysis to exclude doublets. DNA content histograms (PI fluorescence intensity, FL2-A channel) were quantified using FlowJo (version 10.10).

### Subcutaneous xenograft tumor model

Ten BALB/c nude four-week-old mice were procured and housed in a specific pathogen-free environment. They were randomly allocated into two groups of five. UMUC3 cells was selected for constructing the tumor model and the cell number for each mouse was 5 × 10^6 based on preliminary experiments and published protocols [23]. UMUC3 cells stably infected with CPSF3 shRNA and control vector were resuspended at a total volume of 200 µL, consisting of equal parts of PBS and Matrigel. Each mouse received a subcutaneous injection of the cell mixture into the right flank region. Tumor growth was assessed by measuring both length and width with a caliper every 5 days over a 28-day period. Tumor volumes were estimated using the formula:

$$\text{Tumor volume} = 0.5 \times \text{length} \times \text{width}^2 $$                                         

At the 28 days, all mice were euthanized for further analysis.

### RNA sequencing

T24 cells were transfected with siRNA and si-NC for 48 h. Total RNA was extracted from cells using a total RNA extraction reagent (Trizol). Library preparation and mRNA capture were performed using Mustseq and sequencing was carried out on the Illumina platform.

### Identification of DEGs, KEGG analysis and GSEA

Kyoto Encyclopedia of Genes and Genomes (KEGG) pathway analysis and Gene Set Enrichment Analysis (GSEA) was performed to investigate enriched biological themes within gene expression data [24]. “limma” package was used to compare NC group with si-CPSF3 group and screen differentially expressed genes (DEGs) setting a threshold of adjusted p-value < 0.05 and |log2 fold change| >1. Gene sets for GSEA were obtained from the Molecular Signatures Database (MSigDB, http://www.gsea-msigdb.org/gsea/msigdb/index.jsp). “clusterProfiler” package was used to conduct KEGG and GSEA analysis in R with the ranked list of DEGs based on their log2 fold changes, whose function was employed with the following key parameters: nPerm was set to 10,000 for permutation tests, minGSSize and maxGSSize were adjusted to include gene sets containing between 10 and 200 genes, and pAdjustMethod was set to “none”. “enrichplot” package was used to visualize the results, including enrichment plots and dot plots, to highlight the most significantly enriched pathways.

### Statistical analysis

SPSS statistical software (version 20.0.0) and R software (version 4.3.3) were used to perform statistical analyses. The tests were all two-sided. It was considered that p-value of < 0.05 was statistically significant. Patients were randomly separated into training cohort (70%) and internal validation cohort (30%). For continuous variables normally distributed, they were analyzed using the Student’s t-test. For variables not normally distributed, they were analyzed using the Mann-Whitney U test. For categorical variables, they were compared using the Chi-square test or Fisher’s exact test in an appropriate way. “survival” package was used to estimate survival outcomes using Kaplan-Meier method, including OS and DFS. Differences between two groups were evaluated with the log-rank test. “survminer” package was used to determine CPSF3 cut-off values. The cut-off values or the median values of FPKM and H-score was used to dichotomize patients into high and low expression groups and conduct survival and correlation analysis. “coin” package was used to conduct univariate Cox proportional hazards regression, in which variables with a p-value < 0.05 were incorporated in the multivariate Cox regression model. “rms” package was applied to create nomogram and calibration curves and calculate C-index. “timeROC” package was used to create ROC curves. “riskRegression” package was utilized to create DCA curves. “QHScrnomo” package was used to complete k-fold cross validation [25].

## Results

### CPSF3 expression was upregulated in BC and correlated with clinical characteristics

**Fig. 1:**
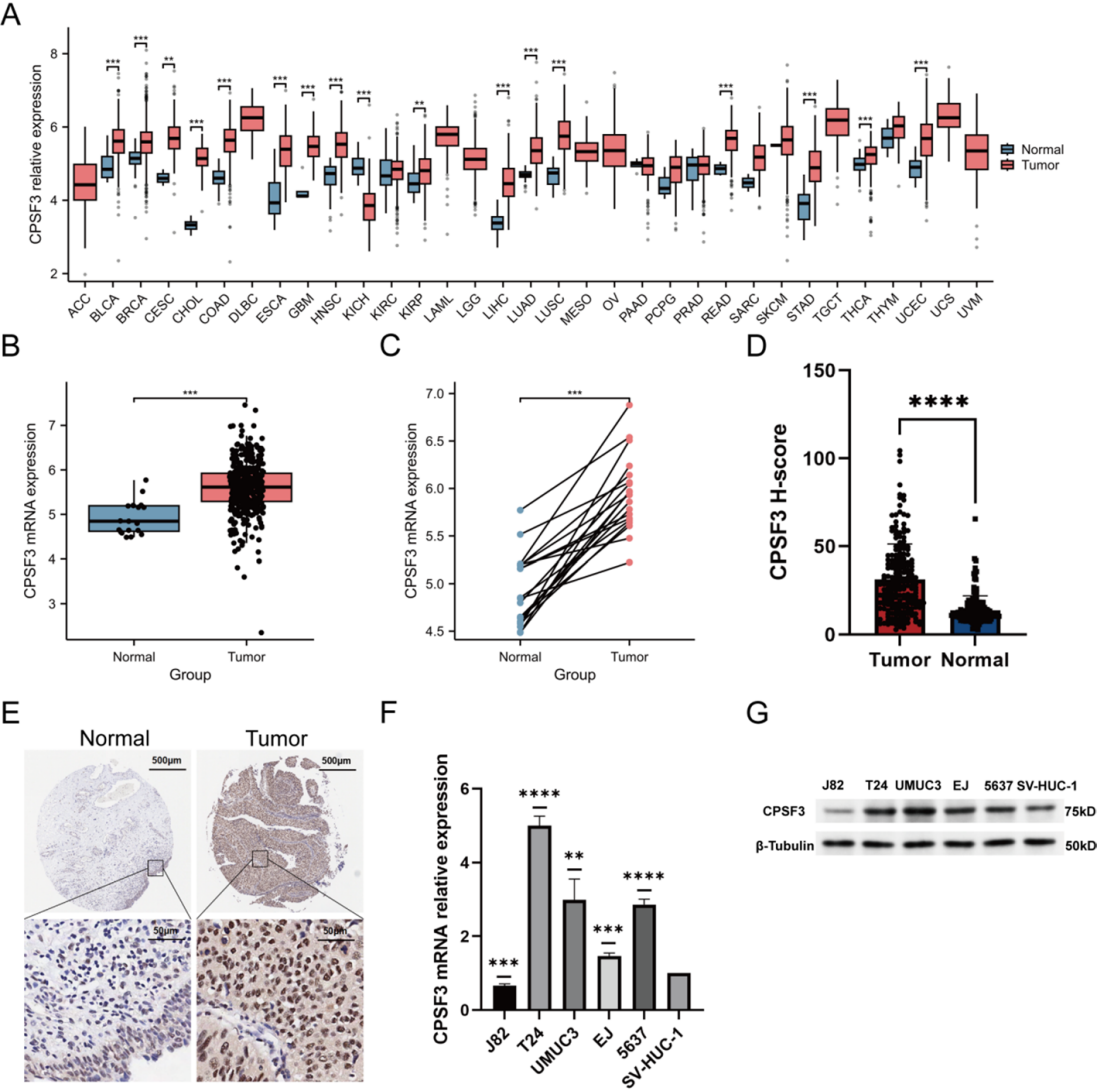
CPSF3 expression in different human tissues. **A** Expression pattern of CPSF3 in 33 types of tumor tissues and para-tumor tissues. **B** Differences in CPSF3 expression between BC tissues and normal bladder tissues. **C** Pairwise difference analysis of CPSF3 in BC tissues and paired para-tumor tissues. **D** Differences in CPSF3 expression between BC samples and para-tumor samples. **E** Representative microphotographs of CPSF3 expression in tumor and para-tumor tissues. (F) CPSF3 mRNA expression in BC and normal cells. **G** CPSF3 protein expression in BC and normal cells. Data of **A**-**C** were collected from the TCGA databases. Data of **D**-**E** were collected from Ruijin cohort. * *P* < 0.05, ** *P* < 0.01, *** *P* < 0.001, **** *P* < 0.0001

First, CPSF3 mRNA expression was analyzed in multiple cancer tissues and their corresponding normal tissues through TCGA database. CPSF3 was upregulated in most of cancer types (Fig. 1A). Then by analyzing TCGA-BLCA datasets, we noticed that CPSF3 expression was significantly higher in BC samples compared with normal bladder tissues (*p* < 0.001) (Fig. 1B). Next, we compared the 19 normal tissues from the same datasets and their paired BC samples, and discovered that CPSF3 was reliably upregulated in BC samples (*p* < 0.001) (Fig. 1C). Whether CPSF3 expression was related to clinical characteristics of TCGA-BLCA datasets were also analyzed. The results showed that CPSF3 expression was significantly elevated in BC samples of high pathology grade compared with BC samples of low pathology grade(*p* < 0.001), while it turned out to be no difference among age, gender and pTNM stage (Figure S1A-F). Then we carried out IHC to investigate CPSF3 expression pattern in Ruijin cohort. There is no statistical difference among H-score and age, gender, pN stage, pM stage, tumor number and tumor size (Figure S1 G-L). Consistent with the result from TCGA-BLCA datasets, H-score was significantly elevated in BC samples compared with para-tumor samples (*p* < 0.0001) (Fig. 1D), and was higher in samples from high grade BC patients than low grade (Figure S1M) via analyzing the correlation between CPSF3 protein expression and clinical characteristics. Besides, H-score was higher in MIBC (T2, T3 and T4 stages) than NMIBC (T1 stage) (*p* < 0.05) (Figure S1N). A BC sample in Ruijin cohort and its paired para-tumor sample were selected and showed in Fig. 1E. CPSF3 was expressed intracellularly, which was consistent with the images in HPA database, as shown in Figure S1O. We conducted qRT-PCR and western blotting analysis to compare the mRNA expression level between the normal uroepithelium cell line (SV-HUC-1) and five bladder cancer cell lines (J82, T24, UMUC3, EJ, 5637). The results showed that CPSF3 mRNA and protein expression was higher in T24, UMUC3, EJ and 5637 than in SV-HUC-1 (Fig. 1F-G). An overview of the TMAs from Ruijin cohort was displayed in Figure S2. These results pointed out that CPSF3 might function as a tumor-promoting factor.

### Gene mutation analysis of CPSF3 in BLCA datasets

**Fig. 2:**
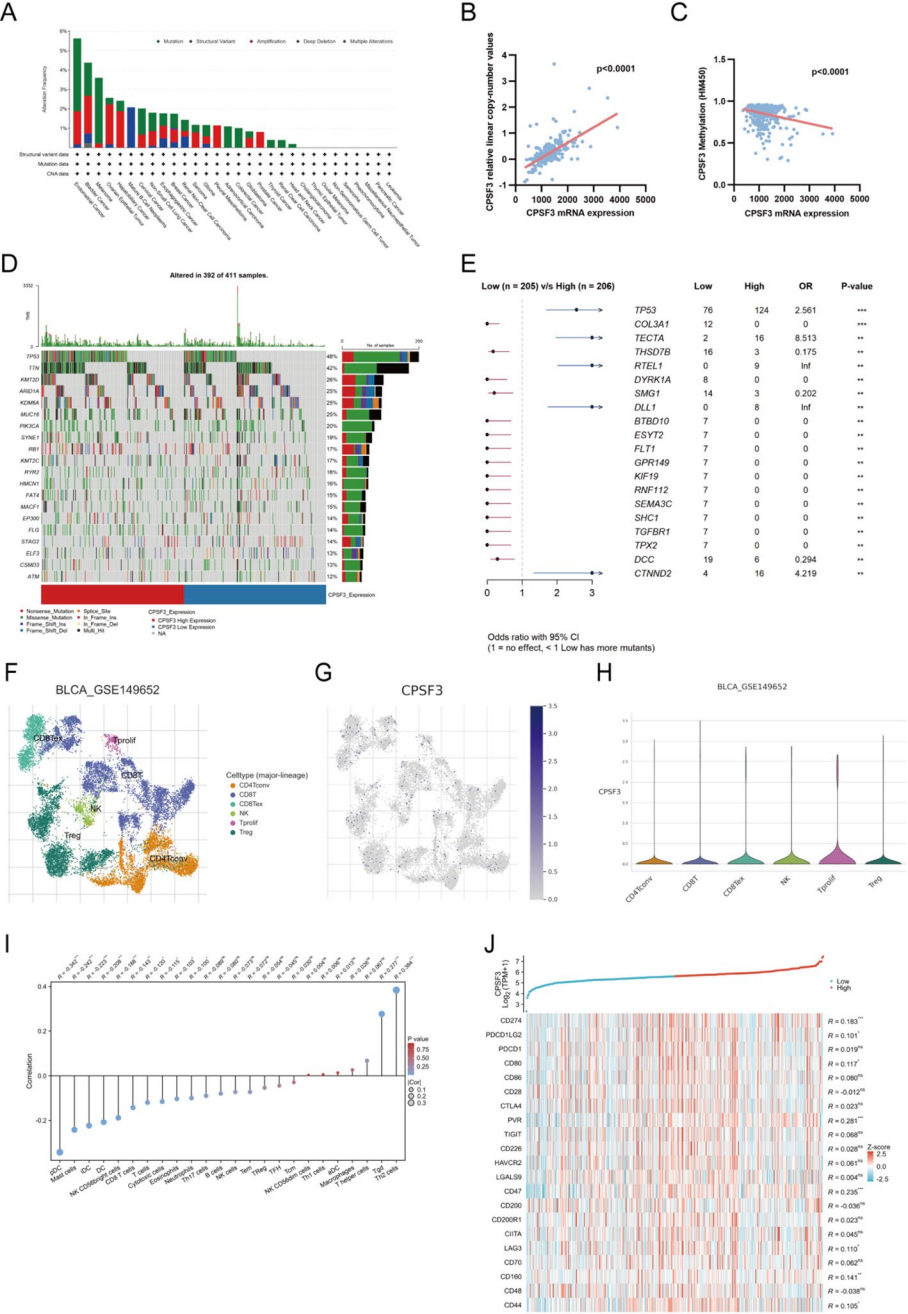
Gene mutation, DNA methylation profiles and distribution in multiple cell types of CPSF3. **A** CNV alteration frequency of CSPF3 in TCGA cancers via cBioPortal database. **B** Correlation between CPSF3 expression and copy-number values. **C** Correlation between CPSF3 expression and promoter methylation. **D** Waterfall plot showing the somatic mutation landscape of CPSF3 in BLCA with top 10 mutation genes displayed. **E** Comparison of gene mutations between CPSF3-high and CPSF3-low groups in BLCA. **F** Major immune cell populations in the TME of BC. **G** The distribution of CPSF3 expression in various immune cell populations. **H** Violin plot displaying CPSF3 expression in various immune cell populations. **I** Correlation between CPSF3 expression and 24 tumor-infiltrating immune cell types. **J** Correlation between CPSF3 expression and common immune checkpoints (ICPs). Data were obtained from the GEO (GSE149652) and TCGA databases. * *P* < 0.05, ** *P* < 0.01, *** *P* < 0.001

We explored the alteration frequency of CPSF3 in multiple cancer types and BC ranked second. Amplification mutation was the main change in BC (Fig. 2A). Subsequently, we investigated the association between CPSF3 mRNA expression and copy number mutation (CNA) as well as methylation in BC. The results turned out that there was a significant positive correlation between CPSF3 expression and CNA and a significant negative correlation between CPSF3 expression and methylation in BC(Fig. 2B-C). Then we analyzed the somatic mutation landscape associated with CPSF3 expression. The waterfall plot showed the mutation rate of the top 20 mutated genes in BLCA datasets and in CPSF3 high expression group, the mutation rate was higher than in low expression group (Fig. 2D). We further investigated the differences of somatic mutations between the two groups. The forest plot showed that genes such as TP53, TECTA, RTEL1 had more mutations in CPSF3 high expression group, and COL3A1, THSD7B, DYRK1A had more mutations in CPSF3 low expression group (Fig. 2E).

### Immune infiltration analysis of CPSF3

Single-cell RNA sequencing data was analyzed and it was confirmed that CPSF3 expressed in multiple immune cell types in TME of BC based on TISCH database, especially highly expressed in Tprolif cells (Fig. 2F-H). Based on BLCA datasets, CPSF3 expression was positively correlated with infiltration level of T gamma delta (Tgd) cells and T helper 2 (Th2) cells, while negatively correlated with infiltration level of plasmacytoid dendritic cells (pDC), mast cells, immune dendritic cells (iDC), dendritic cells (DC), NK CD56^+^ bright cells, CD8^+^ T cells, T cells, cytotoxic cells, eosinophils and neutrophils (Fig. 2I). Further analysis focused on the infiltration level between CPSF3 high-expression and low-expression groups and the result shared the same trend as the former analysis except T cells, cytotoxic cells, eosinophils and neutrophils (Figure S3).

In regard to the relationship between CPSF3 expression level and some of the common immune checkpoints (ICPs), we found that CD274, PDCDLG2, CD80, PVR, CD47, LAG3, CD160, CD44 expression level had significantly positive correlation with CPSF3 expression level (Fig. 2J), indicating the possible impact of CPSF3 expression on the response to immunotherapy.

### High CPSF3 expression predicts poor prognosis

**Fig. 3:**
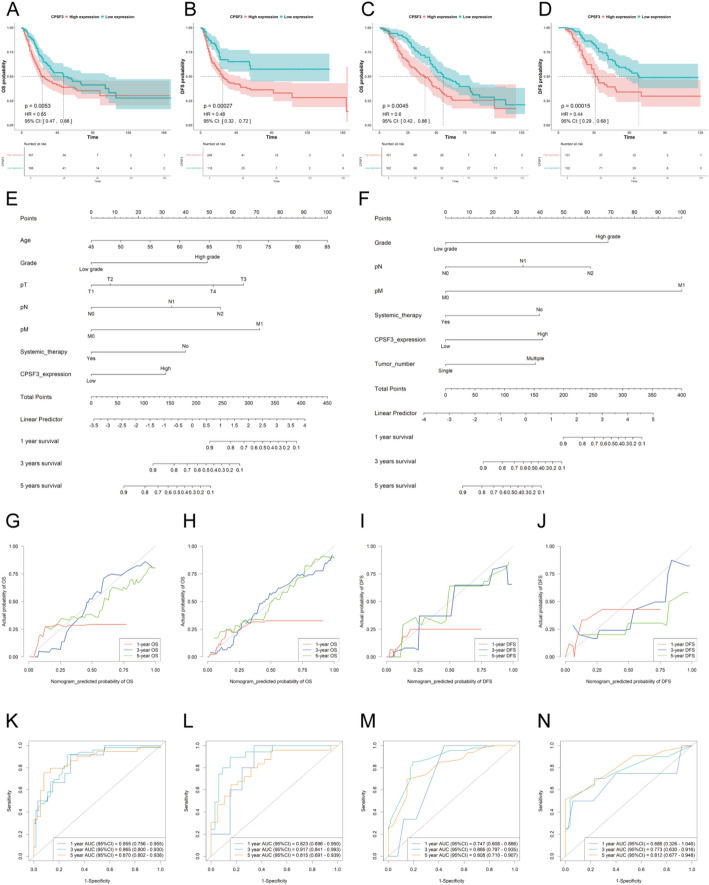
Role of CPSF3 in predicting prognosis of patients from Ruijin cohort and validation. **A**-**B** Correlation analysis between the different expression levels of CPSF3 and OS, DFS in TCGA-BLCA datasets. **C**-**D** Correlation analysis between the different expression levels of CPSF3 and OS, DFS in Ruijin cohort. **E** Nomogram for predicting 1-year, 3-year and 5-year OS. **F** Nomogram for predicting 1-year, 3-year and 5-year DFS. **G**, **H** Calibration curves of 1-year, 3-year and 5-year for OS in the training cohor and the internal validation cohort. **I**-**J** Calibration curves of 1-year, 3-year and 5-year for DFS in the training cohort and the validation cohort. (K)-(L) ROC curves of 1-year, 3-year and 5-year OS in the training cohort and the internal validation cohort. **M**-**N** ROC curves of 1-year, 3-year and 5-year DFS in the training cohort and the internal validation cohort. Data were collected from the TCGA databases and Ruijin cohort

High CPSF3 expression indicated poor prognosis in BC patients from TCGA-BLCA datasets, as BC patients with high expression of CPSF3 were more likely to have worse OS (*p* = 0.0053) and DFS (*p* = 0.00027), when the best cut-off values determining high expression and low expression groups were calculated separately (Fig. 3A-B).

Further analysis aimed to investigate CPSF3’s role in predicting prognosis in Ruijin cohort. Median follow-up period was 40 months (ranging from 1 to 128 months). Patients were separated into high expression group (*n* = 101) and low expression group (*n* = 102) based on the median cutoff value of H-score. We noticed that both OS (*p* = 0.00033) and DFS (*p* < 0.0001) of patients in high expression group was worse by Kaplan–Meier survival analysis (Fig. 3C-D), which also shared similarity with the findings from TCGA-BLCA datasets. Thus, it could shed more light on the prognostic value of CPSF3.

### Univariate and multivariate Cox regression analysis

We randomly separated Ruijin cohort into training cohort (*n* = 142) and internal validation cohort (*n* = 61) by a pre-set ratio of 7:3. Clinical features and CPSF3 expression were summarized and compared, and there was no significant difference between patients of training cohort and internal validation cohort, indicating a comparable baseline (*p* ≥ 0.05) (Table S4). We used training cohort to conduct univariate Cox regression analysis and filter single variant that significantly affecting OS and DFS. Then variants with *p* < 0.05 in the former analysis were included in multivariate Cox regression analysis to exclude confounding variants. Finally, advanced age, high pathological grade, advanced pT, pN and pM stage, lack of systemic therapy, high CPSF3 expression were independently associated with poor OS (Table S5). As supplement, pT2 shared same prognostic value, while pT3 and pT4 led to poor OS compared with pT1. pN2 exhibited no difference, but pN1 showed superior prognostic capability referring to pN0. As for DFS, Grade, tumor number, pN stage, pM stage, systemic therapy and CPSF3 expression were independent prognostic variants associated with DFS, which was not exactly the same as OS (Table S6).

### Nomogram construction for 1-year, 3-year, and 5-year OS and DFS

Depending on multivariate Cox regression analysis, the significantly independent prognostic variants associated with OS and DFS were incorporated in the construction of the nomograms. Age, grade, pTNM stage, systemic therapy and CPSF3 expression were included in OS nomogram. Grade, tumor number, pN stage, pM stage, systemic therapy and CPSF3 expression were included in DFS nomogram. The prediction results for 1-year, 3-year and 5-year OS and DFS were displayed (Fig. 3E-F). From the nomogram, specific values were located for each predictive variant on their respective axes. The scores could be added up from these points to obtain a total score and it could then be mapped to the predicted survival rate at the bottom of the nomogram, thus providing a survival prediction for each patient.

### Internal and external validation and comparison of the nomogram

To demonstrate the predictive value of the nomogram incorporating CPSF3 expression, calibration curves for predicting OS and DFS were shown in Fig. 3G-J. Receiver operating characteristic (ROC) curves were also plotted. The area under the curve (AUC) values of 1-year, 3-year and 5-year OS were 0.855, 0.865, 0.87 in the training cohort and 0.823, 0.917, 0.815 in the internal validation cohort, respectively. Likewise, the AUC values of DFS were 0.747, 0.866, 0.808 in the training cohort and 0.686, 0.773, 0.812 in the internal validation cohort (Fig. 3K-N). The curves indicated that our nomogram had good predictive capability in both the training and the internal validation cohort.

**Fig. 4:**
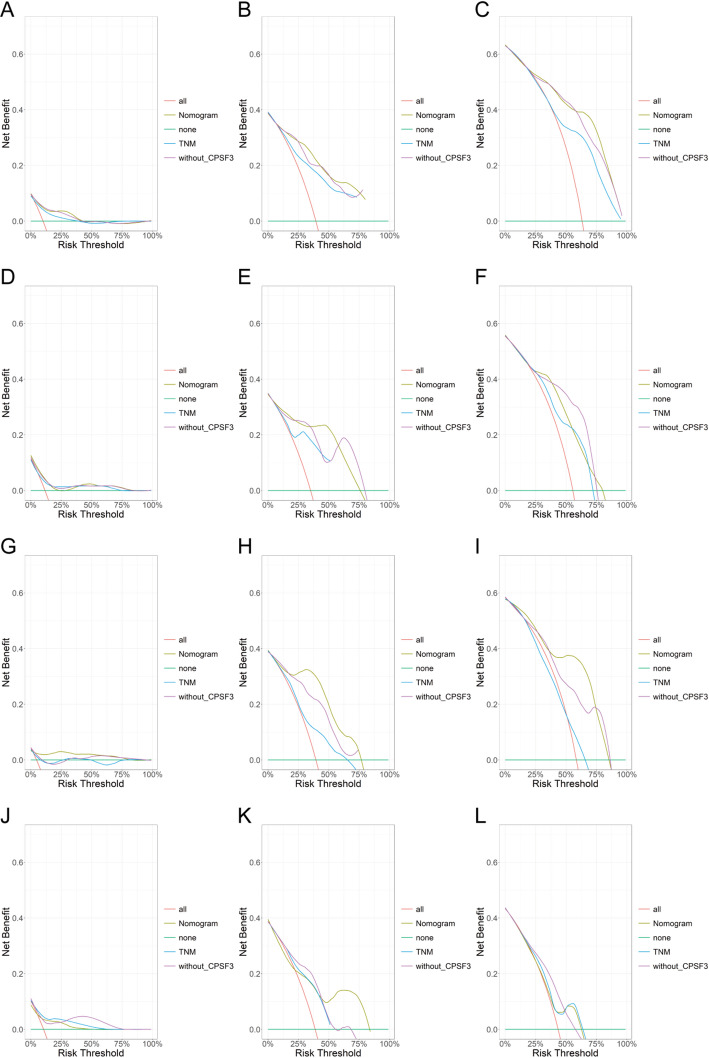
The DCA curves of nomogram and the other two models predicting OS and DFS in Ruijin cohort. **A**-**C** DCA curves of 1-year, 3-year and 5-year OS in the training cohort. **D**-**F** DCA curves of 1-year, 3-year and 5-year OS in the internal validation cohort. **G**-**I** DCA curves of 1-year, 3-year and 5-year DFS in the training cohort. **J**-**L** DCA curves of 1-year, 3-year and 5-year DFS in the internal validation cohort

Next whether our nomogram performed better after including CPSF3 expression than other prediction models remained to be investigated. Based on the training cohort, the other two Cox regression models were constructed. One prediction model included every variant except CPSF3 expression, and another traditional model included just pTNM stage. Concordance index (C-index) was calculated and in our nomogram it was 0.776 and 0.794 for OS in the training cohort and internal validation cohort respectively, while it was 0.706 and 0.706 in pTNM model, and was 0.766 and 0.782 in model without CPSF3 expression. As for DFS, the C-index in our nomogram was 0.768 and 0.742 in the two cohorts, but it reduced to 0.657 and 0.722 in pTNM model and without-CPSF3 model. Decision curve analysis (DCA) curves evaluating our nomogram and the other two models in the training cohort and the internal validation cohort were shown in Fig. 4. The net benefit level of the nomogram was higher than the other two models in most circumstances, which meant that the nomogram had promising and superior clinical application for predicting OS and DFS. These outcomes validated the predictive value of our nomogram and discovered that the predictive performance for OS and DFS was better when incorporating of CPSF3 expression.

Subsequently, BLCA-TCGA datasets were processed as the external validation cohort for predicting OS. Samples with missing data were deleted. Patients were divided into CPSF3 high expression and low expression group based on the median cutoff value of CPSF3 FPKMs in the same way as H-score division. 365 samples were included in the external validation cohort. Similarly, calibration curves, ROC curves, DCA curves were shown in Figure S4. The AUC values of 1-year, 3-year and 5-year OS were 0.747, 0.742, 0.733. The C-index of the nomogram predicting OS was 0.705, while the C-index was 0.626 and 0.69 in the pTNM model and without-CPSF3 model respectively. Since lacking the data of tumor number, the datasets could not serve as the validation cohort for predicting DFS. Given the necessity of external validation for the nomogram, k-fold cross validation was conducted to substitute external validation. With k set 10, the C-index was 0.734. It was concluded that CPSF3 expression actually played a key role in predicting OS and DFS of BC patients.

### Downregulation of CPSF3 inhibits proliferation, clone formation in BC cells and BC tumor growth in vivo

**Fig. 5:**
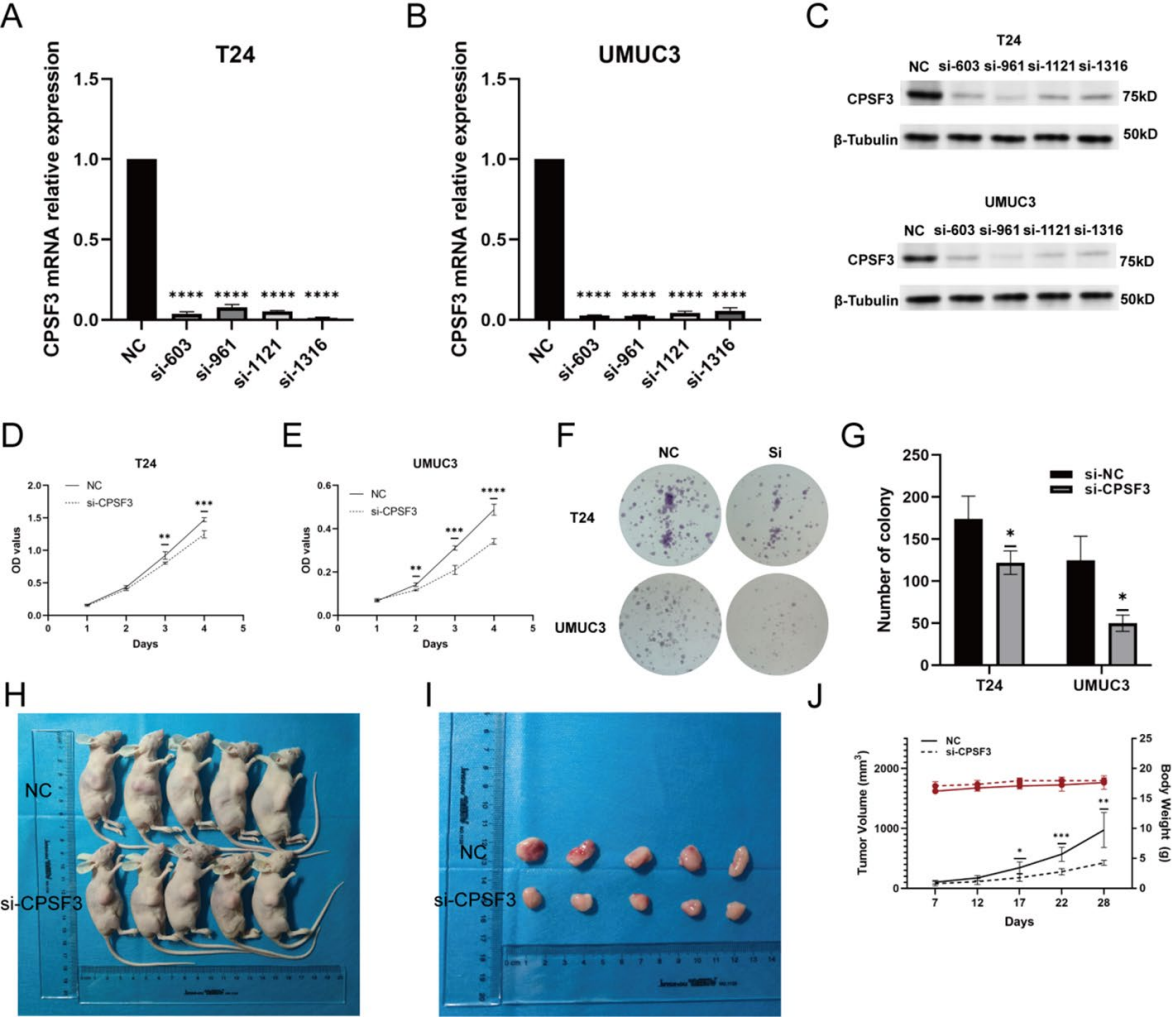
Influence of siRNA on CPSF3 expression and BC cell growth. **A**-**C** Transfection effect of siRNA in T24 and UMUC3 on mRNA and protein level. (D)-(E) CCK-8 assay showing the influence of CPSF3 downregulation on the cell proliferation. **F**-**G** The colony formation assay showing the influence of CPSF3 downregulation on the cell clonogenic capacity. **H** The subcutaneous xenograft tumor models 28 days after UMUC3 cells injection. **I** The dissected subcutaneous tumors in two groups. **J** Body weights and tumor volumes in two groups. * *P* < 0.05, ** *P* < 0.01, *** *P* < 0.001 and **** *P* < 0.0001

Based on the results of qRT-PCR and western blotting, we selected T24 and UMUC3 due to their higher CPSF3 expression for the further in vitro experiment. The four designed siRNA and si-NC was transfected to the two BC cell lines and all the siRNA exhibited significant silencing effect on the mRNA and protein level compared with NC (Fig. 5A-C). The silencing efficiency of each siRNA was assessed and si-961 achieved the greatest reduction in CPSF3 mRNA and protein levels. So we chose si-961 as the ultimate siRNA for next-step cell function assay.

The result of CCK-8 assay proved that downregulation of CPSF3 could suppress BC cell proliferation 2 days after reseeded for UMUC3 and 3 days for T24 (Fig. 5D-E). A significant reduction in colony formation ability in CPSF3 downregulation cells was observed in colony formation assay (Fig. 5F-G).

The subcutaneous xenograft tumor models functioned as in vivo validation of CPSF3’s role. The results showed that the tumors in si-CPSF3 group were significantly smaller than tumors in NC group (Fig. 5H-I). The tumor growth rate was also slower in si-CPSF3 group, while the body weight of the mice had no difference between the two groups (Fig. 5J). These experiments demonstrated that downregulation of CPSF3 inhibited bladder tumor growth both in vitro and in vivo.

### Downregulation of CPSF3 inhibits cell cycle progression in BC cells

**Fig. 6:**
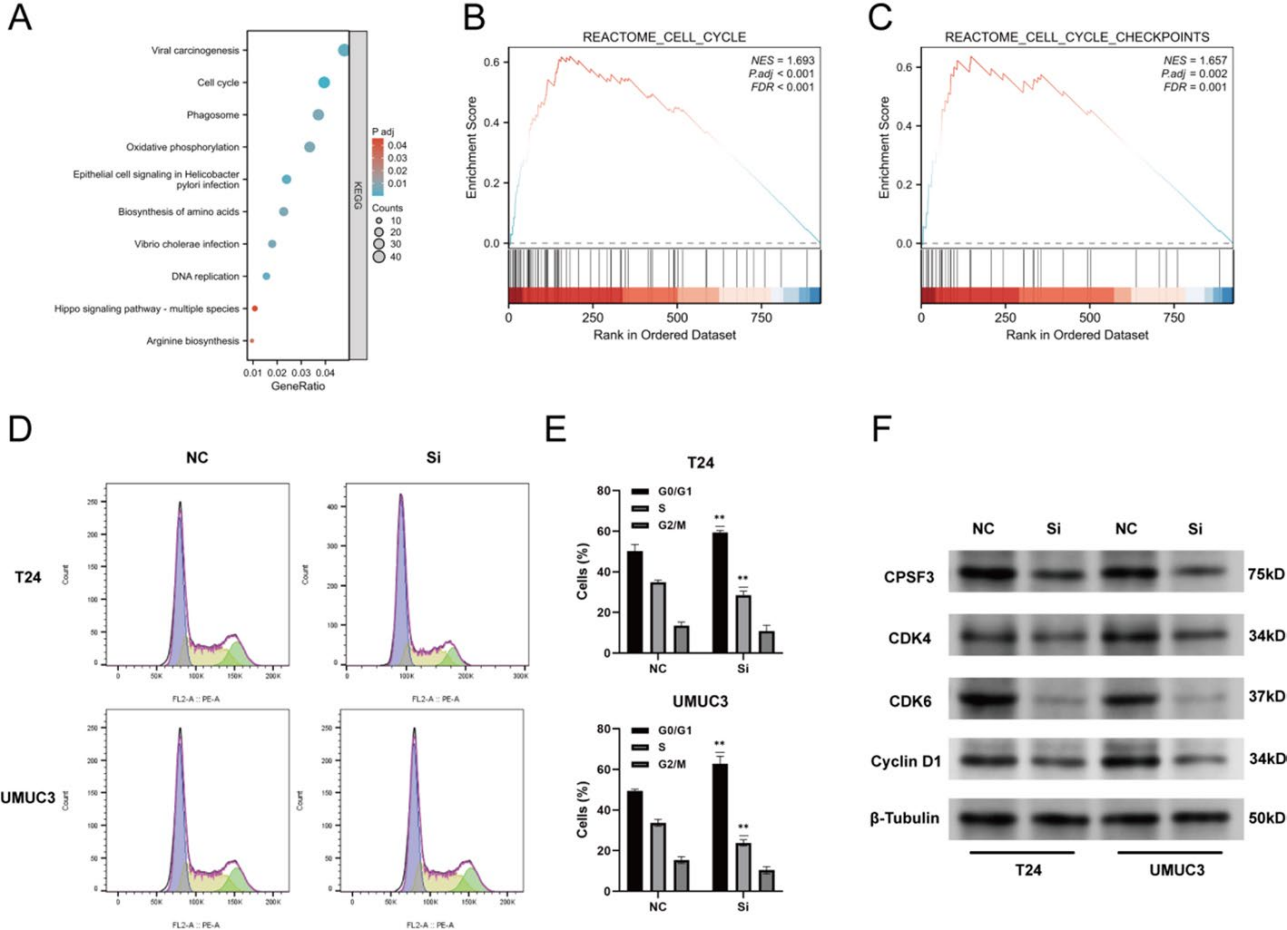
Influence CPSF3 expression on BC cell cycle. **A** Top ten KEGG enrichment pathways. **B** GSEA of cell cycle pathway. **C** GSEA of cell cycle checkpoints pathway. **D**-**E** The cell cycle distribution of T24 and UMUC3 detected by flow cytometry and histograms showng the percentages of each cell cycle phase. **F** CDK4, CDK6 and Cyclin D1 protein expression with altered CPSF3 expression in T24 and UMUC3. * *P* < 0.05, ** *P* < 0.01

Through RNA sequencing, DEGs between si-CPSF3 T24 cells and NC T24 cells were identified and enrichment analysis was further conducted. By KEGG analysis, we noticed that within the pathways DEGs significantly enriched, cell cycle pathway ranked second, which was related to CPSF3’s role on BC cells (Fig. 6A). Next, by GSEA analysis, we found that cell cycle pathway and cell cycle checkpoints pathway were both enriched in CPSF3-high expression phenotype (Fig. 6B-C). These findings indicated that CPSF3 might affect cell cycle progression.

As a result, flow cytometry was employed and demonstrated that downregulation of CPSF3 caused significant S phase reduction and G1 phase accumulation in T24 (*p* < 0.01) and UMUC3 (*p* < 0.01) (Fig. 6D-E). Depending on this finding, we confirmed by western blotting that down-expressed CPSF3 inhibited critical G1/S phase regulator CDK4, CDK6 and Cyclin D1 and then arrested G1/S phase transition (Fig. 6F). It meant that CPSF3 promoted BC development by regulating cell cycle progression.

## Discussion

The CPSF complex is essential in eukaryotic gene expression, primarily involved in the cleavage and polyadenylation of the 3’-end of pre-mRNA, which is crucial for creating mature mRNA molecules ready for translation. The CPSF complex is typically composed of several subunits including CPSF1, CPSF2, CPSF3, CPSF4, and CPSF6, etc., in which CPSF3 provides endonuclease activity that cleaves the RNA [26, 27]. The primary objective of this study was to explore the role of CPSF3 in the progression and prognosis of BC. Our research utilized both TCGA-BLCA datasets and a cohort from Ruijin Hospital to assess CPSF3 expression in BC tissues compared to para-tumor tissues and its relationship with clinical outcomes. The findings revealed that higher CPSF3 expression was significantly and independently associated with worse OS and DFS, as well as higher tumor grades and mutation frequencies. CPSF3 expression influenced the infiltration of various immune cells within the TME. The predictive capability of CPSF3 turned out to be superior than other clinical factors. Besides, the silencing of CPSF3 could inhibit tumor cell proliferation, clone formation and tumor development in vivo. Additionally, CPSF3 high expression phenotype was enriched in pathways correlating with cell cycle. The observed downregulation of CDK4, CDK6, and Cyclin D1 following CPSF3 knockdown suggests that CPSF3 may influence cell cycle progression through modulation of key G1/S regulators.

Our study is the first to identify high tumoral expression of CPSF3 as a negative prognostic variant for OS and DFS of patients with BC after RC. Because of the marked heterogeneity in clinical outcomes among BC patients undergoing RC, there is need for precise prognostic stratification tools. Recent studies have identified several biomarkers that play roles in predicting the prognosis of BC. High plasma level of PTX3, elevated expression of YARS1and mutation of FGFR3 all predicted poor survival probability [28–30]. These outcomes indicate that genetic and molecular biomarkers could offer superior accuracy in prognosis prediction compared to traditional clinical characteristics.

The findings matched those observed in earlier studies, in which the prognositc value of CPSF3 has been confirmed in other cancer types. CPSF3 expression level was dysregulated in tumor tissues and high CPSF3 expression led to unfavorable prognosis in lung adenocarcinoma, hepatocellular carcinoma (HCC) and pancreatic ductal adenocarcinoma (PDAC) from analysis of TCGA database [31–33]. However, incompatible with previous ideas, CPSF3 expression was correlated with only grade in TCGA-BLCA datasets and with grade and T stage in our cohort, while Ning et al. investigated that CPSF3 expression was associated with smoking history, tumor diameter, lymph node metastasis, AJCC stage and radiation therapy in lung adenocarcinoma. We speculated that this might resulted from the different mechanism that CPSF3 influences in different cancer types. Xiong et al. also mentioned the predictive value of CPSF3 in BC based on TCGA [34], which accorded with our observation. Compared with the previous study, the application of IHC including survival and correlation analysis strengthened the fact that CPSF3 predicted poor prognosis of BC patients. The in vitro and in vivo experiment as well as RNA sequencing in our study can further demonstrate the biological function and potential mechanism of CPSF3.

The mechanism of CPSF3 promoting tumor progression has been researched across the cancer landscape. Shen et al.. proved that the blockade of CPSF3 significantly worsened the genomic instability by reducing the expression of genes involved in DNA damage repair, thereby working synergistically with the inhibition of poly (adenosine 5’-diphosphate-ribose) polymerase [35], which could be confirmed by GSEA results in the present study. Besides, Luo et al.. found that CPSF3 was interacted with lncRNA CASC9 to promote its oncogenic effects through the activation of TGF-β signaling in colorectal cancer [36]. Outcomes of other researches indicated that CPSF3 enhances both proliferation and migration in cancer cell lines, such as PDAC and HCC [32, 33], which was also confirmed in BC cell lines in our study. In addition, the potential of therapeutic target of CPSF3 was also under research. Kakegawa et al. found that JTE-607, an anti-leukemia reagent, inhibited the processing of 3-end mRNA within cells. Both the application of JTE-607 and the reduction of CPSF3 levels led to the accumulation of pre-mRNAs and a decrease in the production of inflammatory cytokines, suggesting that the mechanism of action of JTE-607 involves targeting CPSF3 [37]. Mechanistically, CPSF3 facilitates pre-mRNA cleavage and polyadenylation, critical for the maturation and stability of transcripts encoding cell cycle proteins. Dysfunction of CPSF3 could lead to altered processing of these mRNAs, thereby decreasing protein synthesis and resulting in G1 arrest. Additionally, CPSF3 might indirectly modulate cell cycle gene expression by interacting with other regulatory factors involved in cell cycle control. Further investigation of these pathways will be required to fully elucidate the underlying mechanisms.

As lack of multi-center cohort, it could be argued that the prognostic value of CPSF3 still requires larger population to validate. The immune infiltration analysis remain to be validated through immunofluorescence or flow cytometry to detect whether specific immune cells are correlated with CPSF3 expression. The small size of experimental animals constitutes a statistical power limitation and the subcutaneous xenograft model may not fully recapitulate the bladder microenvironment. Future work employing larger animal cohorts and in situ orthotopic models is warranted to further validate these observations.

## Conclusion

A comprehensive surviving and bioinformatics analysis using the TCGA database and our own cohort was performed, as well as in vitro and in vivo experiments. The results provide new insights into the predictive value and biological function of CPSF3 in BC and the possible mechanisms of BC progression. A new model predicting OS and DFS for BC patients after RC was constructed and validated adequately.

## Supplementary Information

Below is the link to the electronic supplementary material.

Supplementary Figure 3

Supplementary Figure 4

Supplementary Figure 1

Supplementary Figure 2

Supplementary Figure Legends

Supplementary Tables

## Data Availability

The public transcriptomic datasets named “TCGA-BLCA” are available in The Cancer Genome Atlas (TCGA) database (https://portal.gdc.cancer.gov/). The genetic alteration features of CPSF3 are available in the cBioPortal database (https://www.cbioportal.org/). The IHC staining images of CPSF3 are available in the HPA database (https://www.proteinatlas.org/). The datasets used to analyze TME status of CPSF3 in diverse cell types named “GSE149652” are available in TISCH2 database (http://tisch.comp-genomics.org/). The RNA sequencing datasets generated and analyzed during the current study are available in the GEO database, named “GSE307514” (https://www.ncbi.nlm.nih.gov/geo/query/acc.cgi? acc=GSE307514). Other data including patient information analyzed during the current study are available from the corresponding author on reasonable request.
