# Supplementary figures and images for: Comprehensive investigation identifies CPSF3 as a novel prognostic and oncogenic biomarker in bladder cancer
Source: Discov Oncol. 2025 Oct 10;16:1847. doi: 10.1007/s12672-025-03672-z (PMC12514100; doi:10.1007/s12672-025-03672-z)

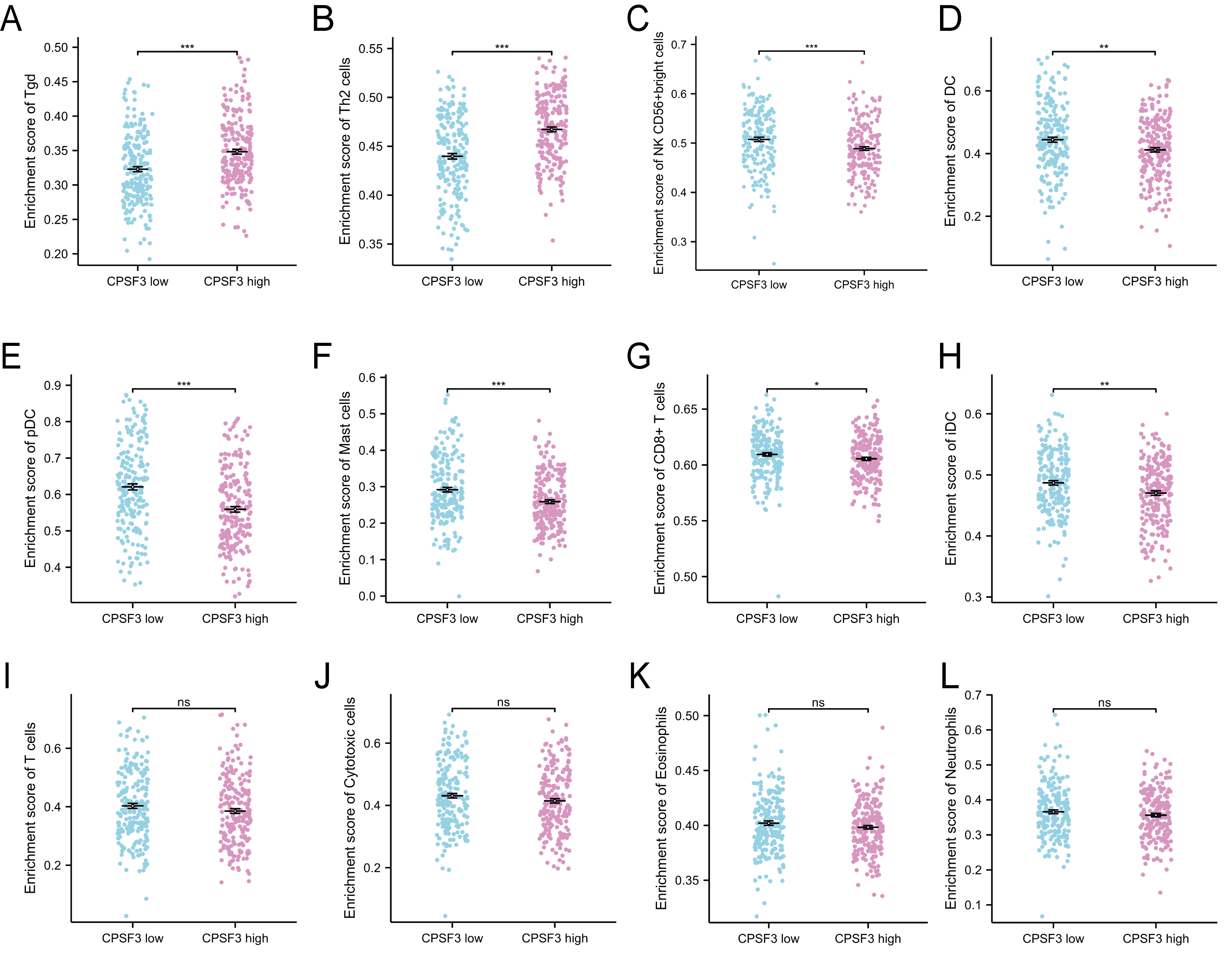

Supplement: Supplementary file 1 — Supplementary Figure 3 [file 12672_2025_3672_MOESM1_ESM.tif]

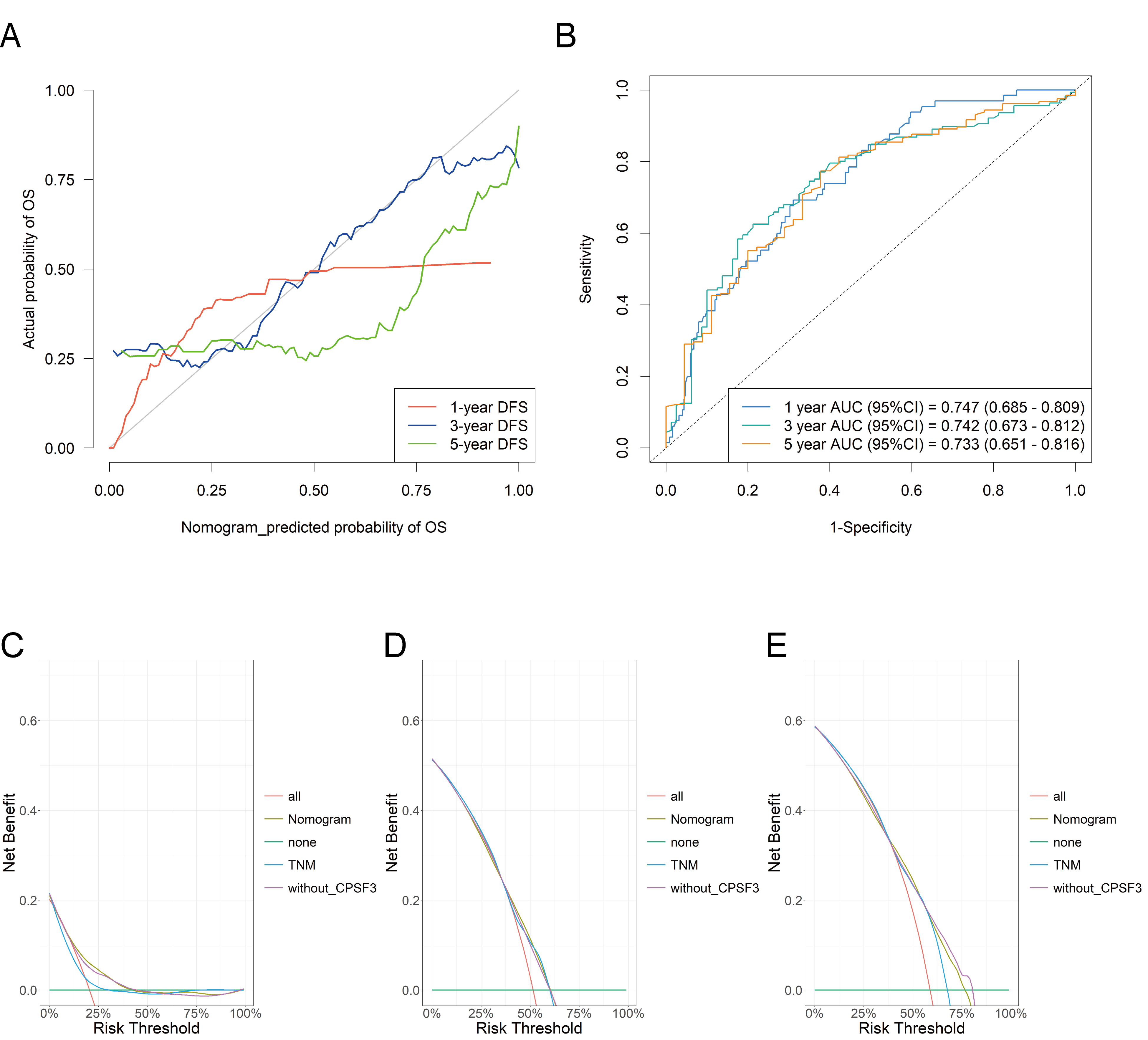

Supplement: Supplementary file 2 — Supplementary Figure 4 [file 12672_2025_3672_MOESM2_ESM.tif]
