# Supplementary material for: Comprehensive investigation identifies CPSF3 as a novel prognostic and oncogenic biomarker in bladder cancer
Source: Discov Oncol. 2025 Oct 10;16:1847. doi: 10.1007/s12672-025-03672-z (PMC12514100; doi:10.1007/s12672-025-03672-z)
Supplement: Supplementary file 5 — Supplementary Figure Legends [file 12672_2025_3672_MOESM5_ESM.docx]

**Figure S1.** mRNA expression and protein expression of CPSF3 in public database and Ruijin cohort. Comparison of CPSF3 mRNA expression in BC patients between male and female (A), ≤65 years and >65 years (B), ≤T1 and ≥T2 (C), N0 and ≥N1 (D), M0 and ≥M1 (E), low grade and high grade (F). Comparison of CPSF3 mRNA expression in BC patients between male and female (G), ≤65 years and >65 years (H), N0 and ≥N1 (I), M0 and ≥M1 (J), single and multiple (K), ≤3 cm and >3 cm (L), low grade and high grade (M), ≤T1 and ≥T2 (N). (O) Images of BC samples from HPA database. * P<0.05, ** P<0.01 and *** P<0.001.

**Figure S2.** Overview and schematic diagram of the IHC images of TMAs. IHC, immunohistochemistry; TMA, tissue microarrays.

**Figure S3.** Correlation between CPSF3 expression and T gamma delta (Tgd) cells (A), T helper 2 (Th2) cells (B), NK CD56+ bright cells (C), dendritic cells (DC) (D), plasmacytoid dendritic cells (pDC) (E), mast cells (F), CD8+T cells (G), immune dendritic cells (iDC) (H), T cells (I), cytotoxic cells (J), eosinophils (K), neutrophils(L).

**Figure S4.** The calibration curves, ROC curves and DCA curves of nomogram predicting OS in the external validation cohort. (A) Calibration curves of 1-year, 3-year and 5-year for OS in the external validation cohort. (B) ROC curves of 1-year, 3-year and 5-year OS in the external validation cohort. (C)-(E) DCA curves of 1-year, 3-year and 5-year OS in the external validation cohort. ROC, receiver operating characteristic; AUC, area under the curve; DCA, Decision curve analysis; OS, overall survival.
