# Supplementary material for: Comprehensive investigation identifies CPSF3 as a novel prognostic and oncogenic biomarker in bladder cancer
Source: Discov Oncol. 2025 Oct 10;16:1847. doi: 10.1007/s12672-025-03672-z (PMC12514100; doi:10.1007/s12672-025-03672-z)
Supplement: Supplementary file 6 — Supplementary Tables [file 12672_2025_3672_MOESM6_ESM.docx]

| **Table S1.** The sequences of siRNAs and primers | |
| --- | --- |
| siRNA/primer | sequence(5'-3') |
| si-603 S | CCGCCAUGUUCAUGAUUGATT |
| si-603 AS | UCAAUCAUGAACAUGGCGGTT |
| si-961 S | GUACCAGACAUAUGUAAAUTT |
| si-961 AS | AUUUACAUAUGUCUGGUACTT |
| si-1121 S | GGCUUAUCCAGAGAAUUAUTT |
| si-1121 AS | AUAAUUCUCUGGAUAAGCCTT |
| si-1316 S | CAGCAAACCAGUGAAUUUATT |
| si-1316 AS | UAAAUUCACUGGUUUGCUGTT |
| CPSF3 F | GCACGTTTACAGCAAGAGGTTGG |
| CPSF3 R | AAGGTTGGCAGTTTTCCCGTCC |
| GAPDH F | GTTCGTCATGGGTGTGAACCA |
| GAPDH R | AGTCCTTCCACGATACCAAAGT |

| **Table S2.** The information of BC cell lines | | | |
| --- | --- | --- | --- |
| Full name | Source | Manufacturer | Catalogue number |
| 5637 | 68-year-old, White male patient | American Type Culture Collection | HTB-9 |
| J82 | 58-year-old, White male patient | American Type Culture Collection | HTB-1 |
| T24 | 81-year-old, White female patient | American Type Culture Collection | HTB-4 |
| UM-UC-3 (UMUC3) | Male patient | American Type Culture Collection | CRL-1749 |
| EJ-1 (EJ) | 43-year-old, White female patient | American Type Culture Collection | CRL-3380 |
| SV-HUC-1 | 11-year-old, male | American Type Culture Collection | CRL-9520 |

| **Table S3.** The information of antibodies | | | |
| --- | --- | --- | --- |
| Antibodies | Dilution ratios | Source | Identifier |
| CPSF3 | 1:500 | ABclonal, China | A2222 |
| CDK4 | 1:1000 | ZENBIO, China | R23888 |
| CDK6 | 1:1000 | ZENBIO, China | R23891 |
| Cyclin D1 | 1:1000 | ZENBIO, China | R380999 |
| β-Tubulin | 1:2000 | Proteintech, China | 10094-1-AP |

| Table S4. Demographics and clinicopathologic characteristics of the cohorts with BC | | | |
| --- | --- | --- | --- |
|  | **Training cohort (N=142)** | **Validation cohort  (N=61)** | **P-value** |
| Age |  |  |  |
| Mean (SD) | 68.1 (9.06) | 68.4 (7.83) | 0.772 |
| Median [Min,Max] | 67.0 [42.0,85.0] | 69.0 [51.0,80.0] |  |
| Gender |  |  |  |
| FeMale | 17 (12.0%) | 7 (11.5%) | 1 |
| Male | 125 (88.0%) | 54 (88.5%) |  |
| Smoking |  |  |  |
| No | 92 (64.8%) | 41 (67.2%) | 0.863 |
| Yes | 50 (35.2%) | 20 (32.8%) |  |
| Tumor_size |  |  |  |
| Mean (SD) | 4.09 (2.21) | 4.34 (1.84) | 0.395 |
| Median [Min,Max] | 3.65 [0.500,15.0] | 4.00 [1.00,10.0] |  |
| Tumor_number |  |  |  |
| Multiple | 26 (18.3%) | 11 (18.0%) | 1 |
| Single | 116 (81.7%) | 50 (82.0%) |  |
| Grade |  |  |  |
| High grade | 122 (85.9%) | 53 (86.9%) | 1 |
| Low grade | 20 (14.1%) | 8 (13.1%) |  |
| Muscle_invasion |  |  |  |
| MIBC | 130 (91.5%) | 50 (82.0%) | 0.083 |
| NMIBC | 12 (8.5%) | 11 (18.0%) |  |
| Vascular_invasion |  |  |  |
| No | 103 (72.5%) | 48 (78.7%) | 0.456 |
| Yes | 39 (27.5%) | 13 (21.3%) |  |
| pT |  |  |  |
| T1 | 12 (8.5%) | 8 (13.1%) | 0.701 |
| T2 | 65 (45.8%) | 26 (42.6%) |  |
| T3 | 44 (31.0%) | 20 (32.8%) |  |
| T4 | 21 (14.8%) | 7 (11.5%) |  |
| pN |  |  |  |
| N0 | 103 (72.5%) | 39 (63.9%) | 0.463 |
| N1 | 24 (16.9%) | 13 (21.3%) |  |
| N2 | 15 (10.6%) | 9 (14.8%) |  |
| pM |  |  |  |
| M0 | 129 (90.8%) | 49 (80.3%) | 0.063 |
| M1 | 13 (9.2%) | 12 (19.7%) |  |
| AJCC_stage |  |  |  |
| Stage l | 12 (8.5%) | 10(16.4%) | 0.251 |
| Stage II | 35 (24.6%) | 14 (23.0%) |  |
| Stage III | 78 (54.9%) | 27 (44.3%) |  |
| Stage IV | 17 (12.0%) | 10 (16.4%) |  |
| Systemic_therapy |  |  |  |
| No | 112 (78.9%) | 47 (77.0%) | 0.918 |
| Yes | 30 (21.1%) | 14 (23.0% |  |
| CPSF3_expression |  |  |  |
| High | 73 (51.4%) | 28 (45.9%) | 0.571 |
| Low | 69 (48.6%) | 33 (54.1%) |  |
| Overall_survival |  |  |  |
| Mean (SD) | 0.599 (0.492) | 0.639 (0.484) | 0.585 |
| Median [Min,Max] | 1.00 [0,1.00] | 1.00 [0,1.00] |  |
| OS_survival_month |  |  |  |
| Mean (SD) | 46.1 (29.3) | 41.3 (26.3) | 0.258 |
| Median [Min,Max] | 41.5 [1.00,128] | 40.0 [4.00,101] |  |
| Progression_free_survival |  |  |  |
| Mean (SD) | 0.528 (0.501) | 0.475(0.504) | 0.494 |
| Median [Min,Max] | 1.00 [0,1.00] | 0 [0,1.00] |  |
| PFS_survival_month |  |  |  |
| Mean (SD) | 39.7 (28.3) | 36.2 (24.4) | 0.374 |
| Median [Min,Max] | 32.0 [1.00,128] | 32.0 [4.00,101] |  |

| **Table S5.** Univariate and multivariate Cox analyses on variables for the prediction of OS of BC patients | | | | | | | |
| --- | --- | --- | --- | --- | --- | --- | --- |
|  | **Univariate** | | | **Multivariate** | | |  |
|  | **HR** | **95%CI** | **P** | **HR** | **95%CI** | **P** |  |
| Age | 1.04 | 1.01 - 1.07 | 0.003 | 1.03 | 1 - 1.06 | 0.0299 |  |
| Gender |  |  |  |  |  |  |  |
| Female | Reference |  |  |  |  |  |  |
| Male | 0.84 | 0.44 - 1.58 | 0.579 |  |  |  |  |
| Smoking |  |  |  |  |  |  |  |
| No | Reference |  |  |  |  |  |  |
| Yes | 0.85 | 0.54 - 1.35 | 0.479 |  |  |  |  |
| Tumor size | 1.03 | 0.94 - 1.12 | 0.533 |  |  |  |  |
| Tumor number |  |  |  |  |  |  |  |
| Multiple | Reference |  |  |  |  |  |  |
| Single | 0.54 | 0.32 - 0.9 | 0.018 | 0.7 | 0.4 - 1.22 | 0.2103 |  |
| Grade |  |  |  |  |  |  |  |
| High grade | Reference |  |  |  |  |  |  |
| Low grade | 0.27 | 0.12 - 0.61 | 0.002 | 0.29 | 0.12 - 0.75 | 0.0099 |  |
| Vascular_invasion |  |  |  |  |  |  |  |
| No | Reference |  |  |  |  |  |  |
| Yes | 1.34 | 0.86 - 2.1 | 0.193 |  |  |  |  |
| pT |  |  |  |  |  |  |  |
| T1 | Reference |  |  |  |  |  |  |
| T2 | 12.7 | 3.01 - 53.64 | 0.001 | 1.89 | 0.42 - 8.42 | 0.4057 |  |
| T3 | 3.73 | 0.89 - 15.61 | 0.071 | 5.83 | 1.29 - 26.35 | 0.0219 |  |
| T4 | 20.82 | 4.67 - 92.79 | <0.001 | 9.72 | 1.9 - 49.76 | 0.0063 |  |
| pN |  |  |  |  |  |  |  |
| N0 | Reference |  |  |  |  |  |  |
| N1 | 1.87 | 1.08 - 3.23 | 0.025 | 2.9 | 1.54 - 5.45 | 0.001 |  |
| N2 | 5.91 | 3.12 - 11.2 | <0.001 | 1.77 | 0.71 - 4.41 | 0.219 |  |
| pM |  |  |  |  |  |  |  |
| M0 | Reference |  |  |  |  |  |  |
| M1 | 6.17 | 3.23 - 11.77 | <0.001 | 2.58 | 1.13 - 5.91 | 0.0247 |  |
| Systemic therapy |  |  |  |  |  |  |  |
| No | Reference |  |  |  |  |  |  |
| Yes | 0.49 | 0.28 - 0.88 | 0.016 | 0.37 | 0.2 - 0.71 | 0.0026 |  |
| CPSF3 expression |  |  |  |  |  |  |  |
| High | Reference |  |  |  |  |  |  |
| Low | 0.43 | 0.28 - 0.67 | <0.001 | 0.52 | 0.32 - 0.85 | 0.0093 |  |

| **Table S6.** Univariate and multivariate Cox analyses on variables for the prediction of DFS of BC patients | | | | | | | |
| --- | --- | --- | --- | --- | --- | --- | --- |
|  | **Univariate** | | | **Multivariate** | | |  |
|  | **HR** | **95%CI** | **P** | **HR** | **95%CI** | **P** |  |
| Age | 1.04 | 1 - 1.07 | 0.029 | 1.03 | 0.99 - 1.07 | 0.1561 |  |
| Gender |  |  |  |  |  |  |  |
| Female | Reference |  |  |  |  |  |  |
| Male | 0.85 | 0.42 - 1.73 | 0.659 |  |  |  |  |
| Smoking |  |  |  |  |  |  |  |
| No | Reference |  |  |  |  |  |  |
| Yes | 1.27 | 0.75 - 2.17 | 0.371 |  |  |  |  |
| Tumor size | 1.08 | 0.97 - 1.2 | 0.169 |  |  |  |  |
| Tumor number |  |  |  |  |  |  |  |
| Multiple | Reference |  |  |  |  |  |  |
| Single | 0.49 | 0.27 - 0.9 | 0.021 | 0.46 | 0.23 - 0.91 | 0.0257 |  |
| Grade |  |  |  |  |  |  |  |
| High grade | Reference |  |  |  |  |  |  |
| Low grade | 0.21 | 0.08 - 0.59 | 0.003 | 0.15 | 0.05 - 0.5 | 0.0019 |  |
| Vascular_invasion |  |  |  |  |  |  |  |
| No | Reference |  |  |  |  |  |  |
| Yes | 0.97 | 0.55 - 1.7 | 0.91 |  |  |  |  |
| pT |  |  |  |  |  |  |  |
| T1 | Reference |  |  |  |  |  |  |
| T2 | 2.8 | 1.03 - 7.6 | 0.043 | 0.52 | 0.17 - 1.6 | 0.2553 |  |
| T3 | 1.6 | 0.62 - 4.15 | 0.333 | 0.74 | 0.24 - 2.31 | 0.6014 |  |
| T4 | 2.63 | 0.87 - 7.89 | 0.085 | 0.48 | 0.13 - 1.88 | 0.2946 |  |
| pN |  |  |  |  |  |  |  |
| N0 | Reference |  |  |  |  |  |  |
| N1 | 1.32 | 0.72 - 2.42 | 0.362 | 2.47 | 1.22 - 5 | 0.0124 |  |
| N2 | 4.1 | 1.93 - 8.72 | 0 | 4.49 | 1.7 - 11.89 | 0.0025 |  |
| pM |  |  |  |  |  |  |  |
| M0 | Reference |  |  |  |  |  |  |
| M1 | 13.41 | 2.73 - 65.93 | 0.001 | 11.84 | 2.07 - 67.71 | 0.0055 |  |
| Systemic therapy |  |  |  |  |  |  |  |
| No | Reference |  |  |  |  |  |  |
| Yes | 0.45 | 0.24 - 0.85 | 0.015 | 0.4 | 0.2 - 0.81 | 0.011 |  |
| CPSF3 expression |  |  |  |  |  |  |  |
| High | Reference |  |  |  |  |  |  |
| Low | 0.47 | 0.28 - 0.79 | 0.004 | 0.42 | 0.23 - 0.77 | 0.005 |  |
